# Factors influencing the quality of acupuncture clinical trials: a qualitative interview of stakeholders

**DOI:** 10.1186/s12906-023-04020-w

**Published:** 2023-09-16

**Authors:** Ying He, Nian Li, Qin Wang, Ying Wang, Zelei Dai, Miaomiao Wu, Haiqi Song, Qian Wen, Ning Li, Yonggang Zhang

**Affiliations:** 1 Division of Internal Medicine, Institute of Integrated Traditional Chinese and Western Medicine, West China Hospital, Sichuan University, Chengdu, China; 2 Department of Medical Administration, West China Hospital, Sichuan University, Chengdu, China; 3 Department of Evidence Based Medicine and Clinical Epidemiology, West China Hospital, Sichuan University, Chengdu, Sichuan China; 4 Division of Head & Neck Tumor Multimodality Treatment, Cancer Center, West China Hospital, Sichuan University, Chengdu, 610041 China; 5 International Medical Center, General Practice Unit, West China Hospital, Sichuan University, Chengdu, China; 6 Department of Periodical Press, West China Hospital, Sichuan University, Chengdu, 610041 China

**Keywords:** Acupuncture, Clinical trial, Qualitative study, Interview, Thematic analysis

## Abstract

**Objective:**

To investigate the influencing factors on the quality of acupuncture clinical trials from the stakeholders, and to provide references for improving the quality of acupuncture clinical trials.

**Methods:**

A qualitative study based on semi-structured interviews was performed. Experts, acupuncturists, editors, and patients were interviewed. The interview results were thematically analyzed from transcribed audio recordings.

**Results:**

A total of 38 stakeholders were interviewed, including 12 experts, 14 acupuncturists, 2 editors, and 10 patients. There were 25 tree nodes and 106 sub-nodes, with 1141 reference points. The key factors influencing the quality of acupuncture clinical trials could be divided into five core theme frameworks: a) trial design, b) trial conduction, c) research results reporting and publication, d) research evidence dissemination, and e) research evidence transformation and application.

**Conclusions:**

The results reveal that to improve the quality of acupuncture trials, it should consider each step of trial design, trial conduction, research results reporting and publication, research evidence dissemination, and research evidence transformation and application. A guideline for quality control of the whole process of acupuncture clinical trials is needed.

## Introduction

Acupuncture has become one of the most widely used forms of traditional medicine in the world [1], and numerous countries and regions endorsed acupuncture therapy and incorporated it into their health systems [2, 3]. The “internationalization” of acupuncture has greatly promoted the development of acupuncture research, with an explosive growth in the number of studies [4]. Since 2010, a number of acupuncture clinical trials were published in high-impact journals such as JAMA, BMJ, and Annals of Internal Medicine [5–7], which provided evidence of acupuncture therapy in clinical practice, and strengthened its international influence [8].

Except for a few high-impact clinical trials, numerous clinical trials could only be published in low-impact journals and some clinical trials were even not published. In the past 10 years, many studies suggested acupuncture might be a potential intervention for diseases, however, only a few conditions were included in practice guidelines [9] and the quality of acupuncture clinical trials was still needed to be improved. Moreover, most clinically effective acupuncture therapies were not supported by high-quality evidence [10]. Several trials in high-impact journals revealed different results [11]. There were still gaps in knowledge transfer between acupuncture trials and acupuncture clinical practice [12, 13]. These factors limited the promotion of acupuncture in clinical practice, and it was urgent to improve the quality.

There have been several methods to improve the quality of acupuncture clinical trials, such as using reporting guidelines, using quality control methods, and performing systematic reviews. Since acupuncture trial was a complex process that spanned a long period of time and involved a large number of people and processes, it was difficult to control the quality [14]. Previously studies only provided opinions from trial’s stakeholders and did not provide opinions from authors’ or other stakeholders. In addition, the previously methods were separately from each other in different aspects. And, no study was conducted in consideration all aspects together, thus, it was needed to perform a new study based on stakeholders and consider the whole process of the acupuncture trials. Thus, we performed the current study, and we hoped the study could help to improve the quality of acupuncture clinical trials.

## Methods

### Study design and ethics approval

**Fig. 1:**
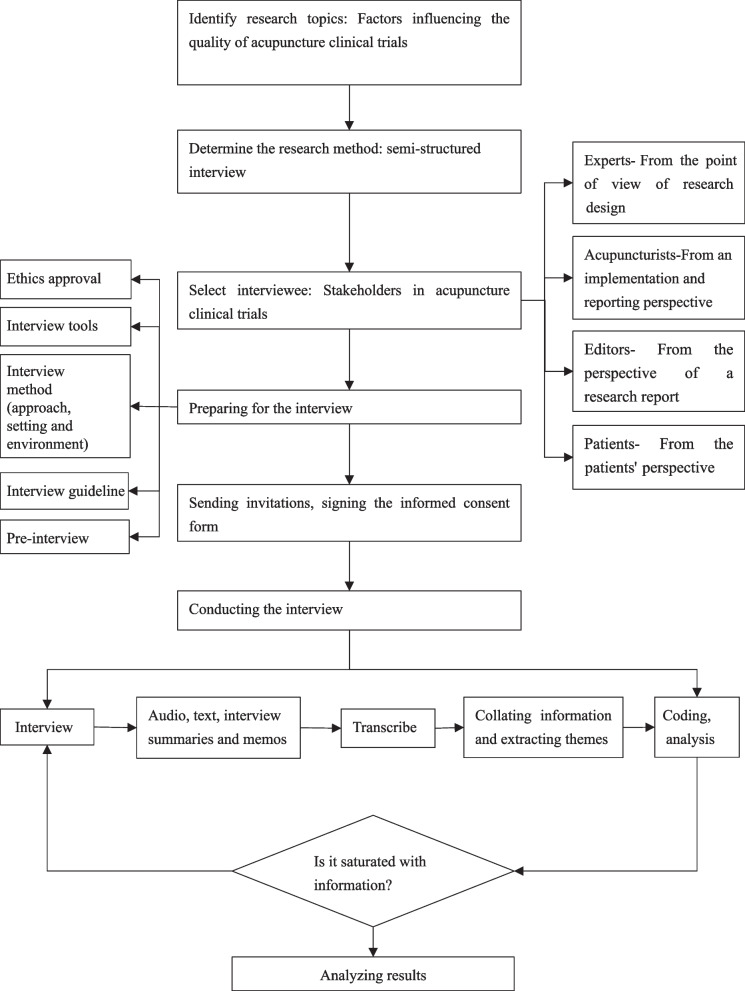
Process of the study

The qualitative study aimed to analyze stakeholders involved in the whole process of acupuncture clinical trials, and thus purposefully sampled with semi-structured interview outlines [15, 16]. The interview conducted from August 2021 to November 2021. The results of interviews were transcribed verbatim then anonymized, and thematic content analysis was performed by NVivo 12 software. The study was reported following the Consolidated Criteria for Reporting Qualitative Research (COREQ) guidelines [17]. The structured process of the study is shown in the Fig. 1. This study followed the Declaration of Helsinki and the four basic principles of medical ethics. Ethical approval was obtained from Ethics Committee on Biomedical Research of West China Hospital of Sichuan University (No. 2021 Annual Review (1188)). Informed consent was obtained from the interviewees who agreed to participate in the study.

### Selection of participants

The inclusion criteria for the interviewees were as follows: stakeholders involved in the quality of acupuncture clinical trials; interviewees consented to record interviews and voluntary signing of informed consent. Exclusion criteria: interviewees who asked to leave in the middle of the interview; those who did not answer all questions; those who gave perfunctory answers. The following four types of stakeholders were interviewed: acupuncture experts (who had participated in acupuncture clinical trials for at least 5 years, and had participated in the design of at least one clinical trial), acupuncturists (who had participated in acupuncture clinical trials for at least 3 years and participated in writing papers related to acupuncture clinical trials), editors (editors of journals related to the publication of clinical trials in acupuncture), and patients (the subjects who had participated in at least one acupuncture clinical trial). Interviewees were also selected based on geography, work background, and etc.

### Research team and reflexivity

The interview was carried out by two postgraduate students (one in acupuncture field and the other in evidence-based medicine field). They were involved throughout the design, optimization, and conduction of the research, and were familiar with all aspects of the research. They were trained in a qualitative research course and had experience in conducting interviews. They were able to extract and interpret the valid information. 

### Sampling and sample size

A stratified purposive sampling method was used to select representatives of different stakeholders for the interview [18]. Because the current study was a qualitative study, the methods of the sample size was consistent with previous studies [19, 20], which meant the recruitment of interviewees ended when the study reached information saturation, which occurred when the interviews with the participants in each role no longer generated new coding information [19, 20]. Otherwise, the interview would continue. 

### Non-participation

Respondents were allowed to withdraw at any time during the process of the research. The researchers recorded the reason for withdrawal and destroyed the recordings and transcriptions accordingly. No one withdrew from the study except that one acupuncturist who declined to be interviewed with the concern that he would not be able to answer the questions well.

### Data collection

The interview was conducted online and face-to-face. Face-to-face interview was conducted with interviewees in Sichuan province, China. It was in a quiet doctor's office and cell phones were turned off during the interview to avoid interference from bystanders as much as possible. Interviewees outside Sichuan province were interviewed online by Tencent Meeting. Depending on the situation, we contacted the interviewees in advance, we sent them an invitation link to the interview, and we explained the background, purpose and significance of the interview, and the format of the interview to them. After obtaining the consent of the interviewees with their signed informed consent form, we sent them the interview guideline and arranged on the time for the interview. For each interview, the interview time, location, and basic information of the interviewees were collected. Interview forms were designed based on the occupations of the interviewees (four types in total). During the interviews, we engaged in conversations with the interviewees regarding the quality of acupuncture clinical trials to establish a broad description of the issue. We asked them each question according to the interview guideline. All interviews were conducted by memos and audio recorded. The information collected from the recording materials was transcribed verbatim within 24 hours after the interview. Interviews were audio taped and transcribed verbatim and all potentially identifiable texts were anonymized, so that participants’ details were kept confidential, and all interviewees were allocated pseudonyms to maintain anonymity.

### Data analysis

The techniques from thematic analysis was used to analyze the data [21]. We analyzed all interview transcription by using qualitative analysis software NVivo 12. Data collection was conducted through semi-structured interviews, as well as field notes and memos [22]. The iterative process continued with data collection, coding, and analysis, followed by further data collection and analysis until saturation was reached, which occurred when the last few interviews fitted existing patterns and did not generate new information. The data analysis was carried out in the following steps: both two researchers firstly read all transcription to familiarize with the data and developed a structured coding tree that started with an open coding. The transcription and open codings were initially coded individually by two researchers. To ensure consistency and reliability of the process, themes were sought, reviewed, defined, and named. In case of disagreement, a third researcher (ZYG) would be consulted to discuss the results until they reached an agreement.

### Quality control of the study

We selected one random respondent from each kind of occupation for a pre-interview before starting the interview, to familiarize with the interview process, revise any issues that arose during the pre-interview, and refine the interview guidelines after discussion the research group. In addition, questions were asked to the interviewees according to a prepared interview guideline, the researcher could be flexible according to the interviewees' responses. In the final stage of interview, participants confirmed what he or she had said, and no new information or themes emerged, so it was decided that enough data had been generated to answer the question. To ensure authenticity, we checked all transcription. We also took notes during and after each interview to record any non-verbal issues which could inform the analysis. We conducted a ‘pre-coding’ exercise to familiarize with the coding process and procedures. Also, to ensure consistency and reproducibility, two researchers coded the interview texts back-to-back, and we invited a third person to consult judgments when disputes arose. The lead researchers oversighted every stage of the analytical process and ensured that findings were representative of the data as a whole.

## Results

### Interviewees’ information

A total of 38 interviewees were included in this study, including 12 experts, 14 acupuncturists, 2 editors and 10 patients. The interview time of experts was 14–56 min, with an average of 37.33 ± 11.72 min, and the interview time of acupuncturists was 17–54 min, with an average of 38.79 ± 8.76 min. Two editors were from the Journal of Acupuncture and Tuina Science and the Chinese Journal of Integrative Medicine, and the interview time was 29 min and 43 min, respectively. The patients’ interview time was between 9 min and 23 min, with an average of 14.80±4.47 min. The interviewees were from Sichuan, Chongqing, Beijing, Shanghai, Jiangsu, Guizhou, Tianjin, etc. The demographics of the participants are shown in Table 1.

**Table 1:** Summary of study participants’ characteristics

		Experts	Acupuncturists	Editors	Patients
Total		12	14	2	10
Gender					
	Male	6	5	/	3
	Female	6	9	2	7
Age (years, mean ± SD)		42.25 ± 8.97	32.14 ± 4.31	47.50 ± 0.71	46.50 ± 13.08
Education					
	Doctoral	10	7	2	/
	Master	/	7	/	3
	Bachelor	2	/	/	/
	Others	/	/	/	7
Professional*					
	Professor	5	/	1	/
	Associate professor	5	1	1	/
	Lecture	1	9	/	/
	Others	1	4	/	/
Interview method					
	Face-to-face	6	10	/	10
	Tencent meeting	6	4	2	/
Interviewed time (min, mean ± SD)		37.33 ± 11.72	38.79 ± 8.76	36.00 ± 9.90	14.80 ± 4.47

*Professor including chief physician; Associate professor including associate chief physician; Lecture including attending doctor; Others were mainly resident doctor

### Thematic analysis of the influencing factors

**Fig. 2:**
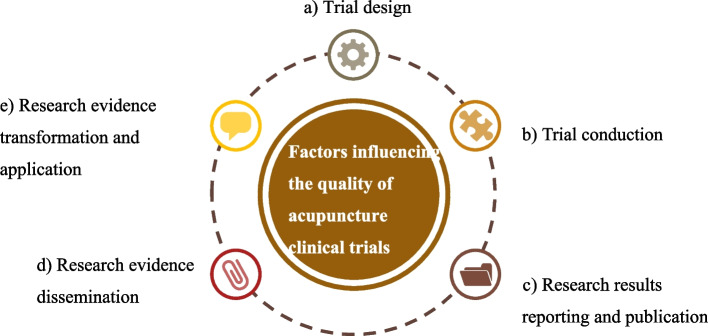
The main themes appeared in the study*. * The key factors influencing the quality of acupuncture clinical trials could be divided into five core theme frameworks: trial design, trial conduction, research results reporting and publication, research evidence dissemination, and research evidence transformation and application

We conducted an open-ended coding of all interview transcripts using qualitative analysis software NVivo 12 line-by-line. It resulted in five main themes, under which 25 tree nodes and 106 sub-nodes were constructed, with 1141 reference point numbers. The main themes appeared in the study is shown in Fig. 2.

The following main themes emerged from the interviewees' sharing of key factors influencing the quality of acupuncture clinical trials during the interviews: a) trial design, b) trial conduction, c) research results reporting and publication, d) research evidence dissemination, and e) research evidence transformation and application. These main themes were interlinked. Some interviewees noted that designing the trial, conducting the trial, reporting and publishing the research findings were the main influencing factors throughout research process, and both trial design and trial conduction were the most important influencing factors.

The tree nodes and sub-nodes under each thematic framework are shown in Table 2. There were 4 tree nodes, 14 sub-nodes, and 366 reference points under the “trial design” theme. There were 9 tree nodes, 50 sub-nodes, and 586 reference points under the “trial conduction” theme. There were 2 tree nodes, 11 sub-nodes, and 103 reference points under the “research results reporting and publication” theme. There were 5 tree nodes, 16 sub-nodes, and 24 reference points under the “research evidence dissemination” theme. 5 tree points, 15 sub-nodes, and 62 reference points were under the “research evidence transformation and application” theme. Brief interview quotations are presented below with unique interviewee IDs.

1. Theme 1: Trial design

**Table 2:** Thematic information on factors affecting the quality of acupuncture clinical trials

Theme	Tree nodes	Sub-nodes	Source of materials	Reference points
Trial design	Design of the trial	Building the research team	12	23
		Formation the research question	8	39
		Designing proposal in a standard	5	7
		Writing the proposal	32	228
		Standardizing acupuncture terminology	1	6
	Conducting pilot trial	Familiar with the process	2	2
		Refining the pilot trial	3	3
	Ethics approval	The factors of researcher	4	5
		The factors of ethics committee	7	11
		The factors of the duration of the trial	1	1
		The factors of the funding	3	3
	Registration	The factors of the researcher	12	35
		Some journals do not uniformly require the registeration	1	1
		The existence of "post-registration"	2	2
Trial conduction	Characteristics of acupuncture	Cost of time-distance for acupuncture	3	4
		Needling sensation response	5	5
		Differences in acupuncture manipulation	1	1
		Specificity of acupoints	2	2
	Research platform and environment	Availability of funding	1	1
		Research platform	2	2
		The factors of the acupuncture treatment environment	2	2
	Coordinated management of the research team	Training & assessment of SOP of the acupuncture trial	10	21
		Selection of researchers	2	2
		Researcher's responsibilities	2	3
		The qualification of the research team	3	3
		Subjective initiative of the researchers	1	1
		The timetable of trial	4	4
		Management of risk of bias	1	1
	The factors of the acupuncture practitioners	Differences in acupuncture practitioners	16	31
		Conduction of the trial	16	38
		Communication between acupuncture practitioners and patients	7	10
		The blinding factors for acupuncture practitioners	7	9
		The standardization of the acupunctureprocedure	7	11
	The factors of outcome evaluator	Truthfulness of data	25	66
		The degree of standardization of data management	5	7
		SOP for outcome evaluation	2	2
		The qualification of the outcome evaluator	18	25
		The blinding factors of outcome evaluator	7	9
		Third-party assessment	3	4
	The factors of statistician	Whether the investigator followed the research proposal	1	1
		Whether the data statistics were standardized	10	14
		Check the integrity of the data	1	1
		Blinded statistican	3	3
	The factors of monitoring	The factor of funding	1	1
		The monitoring was conducted according to SOP	3	3
		The method of monitoring	10	15
		Researcher participant in monitoring	1	1
		Monitoring during trial conduction	11	16
	The factors of trial assistants	Assessment of the treatment compliance in patients	3	4
		The researcher's scientific research capacity and research motivation	2	3
		Conduction of random methods and allocation of concealment	4	5
		Grouping of patients	7	9
		Conduction within of ethical principles	1	1
	The factors of patients	Informed consent of petients	3	6
		The influence of patients on the authenticity of data	16	22
		Expectations effects in patients	10	11
		Patients' preference	7	9
		Patients' variability	4	4
		Patients' compliance	29	155
		The educational levels of patients	3	4
		Patients' cultural context	1	1
		Get support from family and friends	4	5
		The blinding factors of patients	8	12
		The study was ahead of time or delayed	11	16
Research results reporting and publication	The factors of authors	The capacity of scientific research	8	10
		Reporting focused only on the outcome rather than the process	1	1
		Selective reporting of the results	1	1
		Not reporting in accordance with reporting guideline	5	9
		Incomplete reporting of results	19	60
	The factors of journals or editors	Quality control of acupuncture clinical trials by journals	5	5
		Publication bias	3	3
		Long time of peer review	3	3
		Journals could provide guideline for reporting trial design	4	4
		The reporting of the trial was limited by the journals' page number	4	5
		Different journals have different reporting requirements	2	2
Research evidence dissemination	The factors of acupuncture characteristics	The development of acupuncture	1	1
		The characteristics of acupuncture therapy	2	2
		The mechanism of acupuncture was not clear	1	2
		Difficulties with acupuncture manipulation	1	2
	The factors of health policy makers	Resources related to acupuncture were not properly allocated	1	1
	The factors of the research team	Low quality of acupuncture trial	1	1
		Lack of awareness of dissemination of research team	2	2
		The research team do not have sufficient awareness about their aim of acupuncture trial	1	1
		Can't publish high-quality trial in international journal	1	1
	The factors of people who use evidence	Clinicians don't understand acupuncture	1	1
		Clinicians do not adopt research results	4	5
		Acupuncturists in the community do not care about the high-quality acupuncture papers	1	1
	The factors of public	The public don't understand acupuncture	1	1
		Public expectations of acupuncture efficacy	1	1
		The level of trustful of acupuncture	1	1
		Heterogeneity of patients	1	1
Research evidence transformation and application	The factors of transformation platform	Some hospitals lack the conditions for transformation the clinical trial	2	2
		Different environment of research and clinical practice	1	1
	The factors that characterize acupuncture	Differences in genres of acupuncture	2	2
		Characteristics of the discipline of acupuncture	6	11
	The factors of the proposal	The low qualification of the research team	3	6
		Lack of innovation	1	3
		Lack of standardization	2	3
		Low transparency of the whole trial	2	4
		Lack of awareness of advocacy and dissemination among of the research team	5	14
	The factors of research team	Lack of teams that could transform and apply the evidence	2	2
		Lack of awareness of transformation and application	1	1
		Differences in acupuncture practitioner	4	7
		Some acupuncturists don't understand the proposal	1	1
		Transformation and application not strictly following the proposal	3	3
	The factors of patients	Selection and characteristics of patients	2	2

Before an acupuncture clinical trial was conducted, the main task was design the trial, revised and refined it. A rigorous and scientific design was the cornerstone and core of trial. In the acupuncture clinical trial, the initial phase quality of the project depended on ‘designed’, and each aspect and element of the pilot program affected the conduction of subsequent trial [23].

"The second factor that affects the quality of acupuncture clinical trials is the design of the trial, and this is a central influencing factor on the quality. If the trial is not designed properly, it may affect the overall quality." (ID 06-Expert).

(2) Theme 2: Trial conduction

Acupuncture clinical trials were unique and they required both rigorous trial protocols and strict quality control during the conduction phase. It was needed to standardize clinical trials in acupuncture to ensure the authenticity and reproducibility. Whereas there were many factors influencing the conduction phase of acupuncture clinical trials, they could be categorized into the following main areas: Characteristics of acupuncture, research platform and environment, coordinated management of the research team, the factors of the acupuncture practitioners, the factors of outcome evaluator, the factors of statistician, the factors of monitoring, the factors of trial assistants, and the factors of patients.

"The third factor that affects the quality of acupuncture clinical trials is quality control during the conduction of the study after you've designed the trial protocol, and I think it is probably the key to the high quality of the trial." (ID 06-Expert).

"The trial can be designed perfectly, however, it is often not rigorous enough in its conduction, which can easily lead to the separation of design and conduction." (ID 01-Expert).

(3) Theme 3: Research results reporting and publication

The quality control of acupuncture clinical trials was carried out at every process of clinical research, including every step of trial design, conduction, and reporting and publishing the results. It could ensure the credibility of the whole process of acupuncture clinical trials. This study proposed that factors in the reporting and publication of research findings were divided into two main components: authors and editors (or journals).

"There's a big problem with the reporting. For example, some researchers didn't strictly follow the reporting guideline to report the results of study." (ID 09-Expert).

"From the design of the trial to the publication of the paper, it must follow a series of sandards." (ID 01-Editor).

(4) Theme 4: Research evidence dissemination

Acupuncture clinical trials had been developing rapidly in recent years. However, most of the trials were from China, leading to less applicability of the findings when generalized to other populations. The reasons for this situation included the characteristics of acupuncture, health policy makers, research teams, evidence users, the public, and etc.

“First of all, one of the main reasons was that many physicians, especially primary acupuncturists, may not pay much attention to the literatures (even if it was published in JAMA, the New England Journal of Medicine). The fact that the primary acupuncturist has not read the literatures resulted in the ignorance of evidence from these trials, causing him not applying these therapeutic strategies. (I think) it is the main reason(less applicability of the findings when generalized to other populations).” (ID 08-Expert).

(5) Theme 5: Research evidence transformation and application

The ultimate purpose of producing and disseminating evidence was to apply. The transformation and application of high-quality acupuncture evidence was the basis of a new wave of acupuncture clinical trial, and it had guided significance to produce high-quality acupuncture clinical evidence. However, the current lack of generalizability of acupuncture clinical research results had resulted in a low transformation rate of research evidence. The current study identified several factors that influence the transformation of evidence from acupuncture clinical research, including the platform for transformation, the characteristics of acupuncture, the proposal, the research team, and the selection and characteristics of participants (patients).

“Because we have many different schools of traditional Chinese medicine (acupuncture), and there is a lot of variability in different regions, and this may also be one of the reasons why we cannot transform on a large scale.” (ID 06-Expert).

## Discussion

In this study, a qualitative interview was performed to analyze the factors that influence the quality of acupuncture clinical trials. A total of 38 stakeholders were interviewed, the results found that the stakeholders all suggested that quality control should be paid attention throughout the trials, and all steps were important for quality control. The factors could be divided into 5 points, including a) trial design, b) trial conduction, c) research results reporting and publication, d) research evidence dissemination, and e) research evidence transformation and application. Identifying these factors would help improve the quality of acupuncture clinical trials.

Improve quality of acupuncture clinical trials might be different from that in drug trials, because the scientific design was different, and it was also difficult to design the most scientific trials in acupuncture field [23], thus, strengthening the quality was very important [24]. The current study extended the findings of previous studies and highlighted that rigorous, scientific, and standardized study design was the primary guarantee for high quality trial. Therefore, it was needed to standardize the management of Standard Operating Procedure (SOP) of acupuncture clinical trials at all steps. Previous studies reported that design and pre-registration were important strategy to improve quality [25, 26]; in the current study, the stakeholders also suggested that early stage of the trials was very important, and “prevention in advance” would help improving quality of trials.

 Conduction was another key in the whole process of acupuncture trials [27]. Since acupuncture practicing needed technology and personality, acupuncture clinical trials had specific properties, and thus, the quality control in this phase would be different from the drug trials. The quality control should also measure trials’ generality and specialty. The current study suggested a lack of consistent management and training in conduction of acupuncture trials, although previous studies suggested the importance of GCP principles and protocol-specific training [28, 29]. Because, the adaptable of acupuncture intervention in work, the inner motivation of the medical staffs, and the conduction process were all critical, enhance training to improve skills was important for quality of the current stage.

The methodology and quality of reporting were also suggested in the current study to improve the acupuncture clinical trials, which was consistent with previous study in which high-quality research required standardized reporting [30]. However, it did not play the key role from the authors’ aspect [31]. For this aspect, the journals could play more roles, because they could say “no” to papers those did not report well. Therefore, editors should also be crucial in measuring the quality of the acupuncture paper’s “output” [32], which would help improve the quality of acupuncture clinical trials.

The study suggested that dissemination of evidence of acupuncture clinical trials was also an important step, because it facilitated the generalization of results and conclusions into clinical practice [33]. Citation and speed of dissemination were the two main criteria for evaluating the quality of a paper. A high-quality paper represented high-level scientific research [34]. There were many factors which contribute to the quality control, such as the publicity and promotion of the research team, the allocation of research resources by health policy makers, and the trust of users of evidence and the public. Thus, the dissemination of evidence of acupuncture trials should provide more attention for these factors. Evidence transformation and application was the ultimate ‘measure’ of the quality of acupuncture clinical trials, as well as the source and reference for new studies [35]. The transformation and application of research evidence was of great significance in clinical practice [36]. Currently, there were known gaps between trials and clinical practice [37], and the reasons included the research contexts, selection and characteristics of participants, the conversion platform, and investigators, and low quality of evidence [10]. Thus, it was also needed to focus on the research evidence transformation and application in future.

There were several strengthens of the study. First, using a qualitative method was the major strength. Second, stakeholders in different locations and disciplines provided more reliable results. Third, the interviewers had experience and training in conducting qualitative research and strictly followed the research steps and methods. However, there were also several limitations. First, inherently subjective was  the main problem. Second, international experts were not involved in the current study. Third, the sample size was  relatively small, and future studies should be performed to validate these results.

In conclusion, the results reveal that to improve the quality of acupuncture, trial design, trial conduction, research results reporting and publication, research evidence dissemination, and research evidence transformation and application should be all considered. Thus, it is needed to develop a guideline for the quality control of the whole process of acupuncture clinical trials.

## Data Availability

Statement Data are available upon reasonable request from corresponding author. Study protocol and original data are available on request by emailing the corresponding authors.

## Abbreviations

JAMA: The Journal of the American Medical Association
BMJ: British Medical Journal
COREQ: Consolidated Criteria for Reporting Qualitative Research
SOP: Standard Operating Procedure
CONSORT: Consolidated standards of reporting trials
GCP: Good Clinical Practice

## Declarations

### Other information

The study is the basis to develop a guideline, and the guideline was registered on the Global Practice Guidelines Registry Platform (registration number: IPGRP-2021CN093).
